# Scope of Microbial Transglutaminase for Site-Specific and Oriented Immobilization of Native Antibodies from Various Host Species

**DOI:** 10.1021/acs.langmuir.5c06485

**Published:** 2026-02-27

**Authors:** Emily Beitello, Kwame Osei, Faith E. Breausche, Jon A. Friesen, Jeremy D. Driskell

**Affiliations:** Department of Chemistry, 6049 Illinois State University, Normal, Illinois 61790, United States

## Abstract

Modification of antibodies to chemically couple labels or immobilization reagents is essential for developing biosensors. Typically, conjugation occurs through chemical methods that leverage reactive amines and thiols on native antibodies; however, this nonspecific approach can interfere with antibody function. Microbial transglutaminase (mTG) is an enzyme that has been used for site-specific conjugation of chemical modifiers to the F_c_ region of native antibodies, but thus far mTG-mediated conjugation has been limited to production of antibody-drug conjugates with human IgGs. Here, we assessed the scope and versatility of mTG to target IgGs, with the goal of site-specific conjugation to facilitate oriented immobilization. A fluorescently labeled peptide was conjugated to several IgG host species and subclasses commonly used to produce monoclonal (e.g., mouse IgG1 and rat IgG1) and polyclonal (e.g., rabbit IgG and goat IgG) antibodies. SDS-PAGE confirmed site-specific conjugation of the peptide to each of these IgG subclasses. In addition, NH_2_–PEG_4_-biotin was chemo-enzymatically installed on the F_c_ region of each tested IgG, as confirmed by Western blot analysis. Site-specific biotinylated antibody was immobilized on a streptavidin-coated substrate to evaluate antigen binding activity in a functional assay. The site-specific conjugation of biotin enabled the formation of an oriented capture antibody layer to enhance antigen binding when compared to the performance of a functional assay constructed by immobilizing a randomly biotinylated antibody prepared by conventional chemical conjugation. These results highlight the broad scope of mTG to site-specifically conjugate native antibodies to improve analytical performance of biosensing platforms.

## Graphical abstract

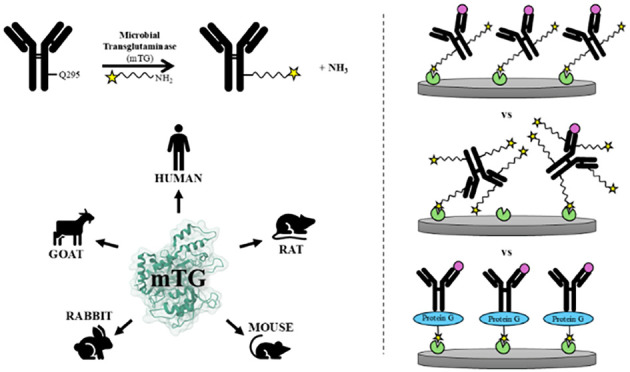

## Introduction

Chemical functionalization of antibodies is essential for many established and emerging biomedical and bioanalytical technologies. Conjugation of therapeutic drugs, chemical tracers, reporter molecules, and chemical immobilization linkers to antibodies facilitates treatment, imaging, and sensing for therapeutic and diagnostic biotechnologies. Conventional coupling to antibodies typically relies on chemical methods, leveraging the reactivity and abundance of primary amine functional groups located on the side chain of lysine residues.^1–3^ However, chemical modification is often difficult to control resulting in heterogeneous antibody conjugates that are challenging to reproduce and characterize.^4,5^ Homogeneous modification, with respect to the number, site, and reproducibility of modifications, is highly desirable. Moreover, chemical moieties are ideally installed on the F_c_ region of an IgG antibody to limit impact on the antigen binding site and overall biological activity of the antibody.^6–11^ This is particularly relevant to surface functionalization when orientation of immobilized antibody governs analytical performance by reducing nonspecific binding and increasing the density of antigen binding sites.^12–18^ Thus, there is a continued effort to develop robust and reproducible site-specific conjugation strategies that are critical to the success and optimal performance of downstream applications.

Several site-specific conjugation strategies have been reported that offer improved properties and function compared to nonspecific (i.e., random) methods.^5,19–21^ Protein engineering approaches allow insertion of short peptide sequence tags, integration of unnatural amino acids, or incorporation of bioorthogonal chemical functional group into proteins, such as antibodies.^22–25^ This allows for highly controlled introduction of chemical handles to efficiently and reproducibly couple drug molecules, labels, or immobilization linkers. Nevertheless, protein engineering is labor- and time-intensive; every antibody requires a unique production process. Consequently, protein engineering for site-specific antibody conjugation is primarily limited to antibody-drug conjugates, in which a single antibody with high specificity and affinity is identified for conjugation to a drug and mass produced.

A more generalizable approach is required for the site-specific modification of antibodies employed in biosensors and immunoassays. Capture antibodies are immobilized on solid supports (e.g., surfaces or nanoparticles), and it is well-established that the orientation of the immobilized antibody impacts antigen capture efficiency.^14–18^ Thus, site-specific conjugation of a chemical linker to facilitate oriented antibody immobilization can potentially improve assay performance. Detection antibodies are conjugated to reporter molecules (e.g., fluorophore), and nonspecific labeling of the antibody can lead to diminished binding affinity and variable degree of labeling. However, it is not realistic to recombinantly express the numerous detection and capture antibodies used in immunoassays, and there is a need for site-specific conjugation methods that are broadly applicable to off-the-shelf commercial antibodies for assay development and optimization.

Directed chemical conjugation is an alternative site-specific approach that is compatible with native antibodies and does not require recombinant engineering. One such strategy is to partially and selectively reduce conserved interchain disulfide bonds in human IgGs and to subsequently modify using maleimide chemistry.^26–30^ However, controlled reduction is crucial and can lead to the unwanted reduction of intrachain disulfides to destabilize the antibody and the production of heterogeneous modifications. Moreover, maleimide chemistry is reversible,^31,32^ although ThioBridge, a strategy to reduce disulfides and incorporate drug molecules during a rebridging step, overcomes some limitations and is being commercially developed.^26,31^ While selective reduction of disulfides has primarily focused on antibody-drug conjugation, given the compatibility to modify native antibodies, this method has also been explored for site-specific immobilization of capture antibodies in a biosensor.^16^ Full reduction of antibody interchain disulfide bonds to produce two identical fragments composed of a single light and heavy chain has been reported for site-specific and oriented immobilization on gold surfaces via Au–S chemisorption. This approach was used to immobilize a capture antibody in a biosensor and found to enhance assay performance,^16^ although precise reduction of only interchain disulfides is difficult to achieve, conditions vary with antibody subtype, and reduced fragments are susceptible to aggregation.^33^ A second approach exploits F_c_ binding peptides as a templated-directed strategy to regioselectively modify an antibody.^34–36^ These methods tether a lysine-reactive payload to an F_c_ binding peptide. These constructs ensure that the chemical modifier is installed on the F_c_, in contrast to conventional lysine-reactive modifiers that are installed on all accessible lysine residues distributed throughout the antibody structure. While templated-directed strategies are emerging as a promising technique for localized conjugation to antibodies, it requires expertise to engineer F_c_ binding peptides and to chemically synthesize unique payloads. Thus, this approach is less suitable for routine site-specific modification of antibodies with widely available reagents.

Chemo-enzymatic methods are another alternative to protein engineering for site-specific modification of native antibodies. Exploitation of conserved glycans on the F_c_ region of IgG antibodies has resulted in efficient site-specific conjugation that is generally applicable. Glycan-mediated conjugation typically requires enzymatic trimming of the native antibody glycan, followed by enzymatic installation of a chemical modifier using a transferase.^37–45^ The scope of glycan-mediated site-specific conjugation has been explored and used to successfully produce IgG conjugates derived from several species; however, it has been predominantly applied to human IgG for antibody-drug conjugate development.^46^ While a promising approach, it has been noted that the variety of glycoforms can lead to conjugate heterogeneity and variable conjugation efficiency has been observed for different payload chemistries.^25,47^ A second chemo-enzymatic approach employs microbial transglutaminase (mTG) to selectively target a privileged glutamine residue (e.g., Q295) in the IgG heavy chain.^48–52^ mTG catalyzes the formation of an amide bond between the γ-carboxamide group of the glutamine side chain and primary amine reagents. mTG-mediated conjugation has been extensively studied to synthesize antibody-drug conjugates; however, studies have been limited to human IgGs for the development of antibody-drug conjugates. mTG is underexplored for conjugation to IgG derived from other species (e.g., mice, rats, goats, rabbits, etc.) that are typically the source of capture and detection antibodies used in biosensors and immunoassays.

Our group previously reported on mTG-mediated site-specific conjugation of biotin to orient an antibody.^53^ However, that work was limited solely to the site-specific modification of a rat IgG antibody. Here, we aim to better define the scope and limitations of mTG-mediated conjugation by systematically investigating the versatility of mTG to site-specifically modify native IgGs derived from various host species. The Q295 modification site is reported to be conserved among several IgG host species and subclasses commonly used to produce monoclonal (e.g., mouse IgG1 and rat IgG1) and polyclonal (e.g., rabbit IgG and goat IgG) antibodies. Therefore, we selected commercially available IgGs derived from these species to assess mTG-mediated, site-specific conjugation as an effective means for controlled and oriented immobilization. First, a fluorophore-labeled peptide was conjugated to each IgG molecule. These studies establish mTG catalyzes the site-specific installation of the fluorescent probe on the heavy chain and serve as a model system in the development of a detection antibody for use in a biosensor. Next, a biotin moiety was installed on the IgG heavy chain of the IgGs to enable oriented immobilization on a streptavidin functionalized surface. We confirm site-specific biotinylation of IgG heavy chains, for IgGs with the conserved Q295. Data confirm that antigen binding was enhanced in immunoassays developed by immobilizing site-specific biotinylated antibodies, when compared to chemically (e.g., randomly) biotinylated antibodies or protein G mediated immobilization of native antibodies. Importantly, this work demonstrates the scope of mTG for site-specific modification of native antibodies that is essential to synthesize antibody functionalized surfaces in advanced biosensors.

## Experimental Section

### Materials and Reagents

The following IgGs were purchased from Bio X Cell: human IgG1 isotype control (catalog #BE0297), human IgG2 isotype control (catalog #BE0301), mouse IgG1 isotype control (catalog #BE0083), and rat IgG1 anti-horseradish peroxidase (catalog #BE0088). Mouse IgG1 anti-horseradish peroxidase monoclonal antibody was purchased from Thermo (catalog #MA1–10371). Mouse IgG2b anti-horseradish peroxidase monoclonal antibody was purchased from MyBioSource (catalog #MBS531597). Microbial transglutaminase (mTG) was purchased from Zedira (catalog #T300). A PNGase F deglycosylation kit was purchased from New England Biolabs (catalog #P0704L). The following custom peptides were purchased from AAPPTec: dansyl-KKCC–COOH and dansyl-KKKCCC–COOH. Magne Protein G Beads were purchased from Promega. Amino-poly­(ethylene glycol)-biotin (NH_2_–PEG_4_-biotin) was purchased from BroadPharm (catalog #BP-22115). *N*-hydroxysuccinimide ester-poly­(ethylene glycol)-biotin (NHS-PEG_4_-Bt) was purchased from Pure PEG (catalog #246904–250L). The following items were purchased from Thermo Fisher Scientific: EZ-Link NHS-PEG_4_ Biotinylation Kit (catalog #21455), phosphate buffered saline (PBS) packets, horseradish peroxidase (HRP) (catalog #31490), ammonium persulfate, glycine, tris­(hydroxymethyl)­aminomethane (Tris base), tris­(hydroxymethyl)­aminomethane hydrochloride (Tris HCl), methanol, potassium chloride, streptavidin-HRP (catalog #434323), 1-Step Ultra TMB-Blotting solution, PVDF/filter paper sandwich 0.2 μm pore size, streptavidin coated clear 96-well plates, biotinylated recombinant protein G (catalog #29988), Corning low binding microcentrifuge tubes, and 1-Step ABTS substrate solution. Tween-20, bovine serum albumin (BSA) (catalog #A8806), sodium dodecyl sulfate (SDS), and acetic acid were purchased from Sigma-Aldrich. Acrylamide/bis-acrylamide (37.5:1) was purchased from Research Products International. Precision Plus Protein Dual Color Standards protein ladder was purchased from Bio-Rad.

### Synthesis of Peptide-Antibody Conjugates

The IgG antibodies were deglycosylated to remove bulky glycans at N297 prior to mTG conjugation using PNGase F deglycosylation kit. Following the manufacturer’s protocol, 100 μL of 1 mg/mL antibody, 10 μL of glycobuffer 2 (10×), and 2 μL of PNGase F were added to a low binding microcentrifuge tube and mixed gently by pipetting up and down. The deglycosylation reaction incubated at 37 °C for 24 h. Immediately following deglycosylation, mTG conjugation was performed. Fluorescently labeled peptides were added into the reaction mixture to generate a 60-fold excess of peptide to antibody. Lastly, 3 μL of 320 U/mL mTG was added into the reaction mixture for a final mTG concentration of ∼7 U/mL. The conjugation reaction incubated at 37 °C for 24 h.

### SDS-PAGE of Peptide-IgG Conjugates

UV images of SDS-PAGE gels were taken to verify site-specific conjugation of the fluorescently labeled peptides onto the IgG heavy chain. IgGs were chemically reduced into heavy and light chain fragments then separated in a 12% SDS-PAGE gel. Native antibodies and peptide conjugated antibodies were reduced with a DTT loading buffer (4×) by mixing at a 3:1 (v/v) ratio of antibody to loading buffer. This mixture was incubated at room temperature for 30 min with periodic vortex mixing, then placed in a hot water bath (∼95 °C) for 5 min immediately prior to loading approximately 2.5 μg of IgG in each lane. A Bio-Rad Precision Plus Protein Standards ladder with fluorescent bands at 25 and 75 kDa served as a molecular weight marker. The loaded gel was placed in running buffer (250 mM glycine, 25 mM Tris base, and 0.1% SDS) and electrophoresed at 150 V for less than 1 h or until the loading dye reached the bottom of the resolving gel. The gel was imaged at 365 nm with a UVP GelDoc-It^2^ Imager. After UV imaging, the gel was stained overnight in a Coomassie solution (45% methanol, 10% acetic acid, and 0.1% Coomassie Blue), followed by destaining for 12 h in a 45% methanol and 10% acetic acid solution.

### Synthesis of Biotin-IgG Conjugates

#### Site-Specific

IgGs were site-specifically biotinylated via mTG conjugation to the heavy chain Q295. To improve conjugation efficiency, IgGs were first deglycosylated as previously described. Immediately following deglycosylation, mTG conjugation was performed by adding 40-fold excess of NH_2_–PEG_4_-biotin and ∼7 U/mL mTG and incubating at 37 °C for 24 h.

Protein G magnetic beads were used to isolate biotinylated IgGs from the reaction mixture, removing excess biotin reagent, mTG, and PNGase F. Following the manufacturer’s protocol, 50 μL of Promega Magne Protein G Beads were added to a low binding microcentrifuge tube and placed into a magnetic stand to allow the separation of the magnetic beads from the supernatant. The storage buffer was removed from the tube and discarded. The separated beads were resuspended in 500 μL of 1% BSA in 10 mM PBS and incubated for 15 min in an automated vortex mixer at 1300 rpm. After blocking, the beads were magnetically separated to remove the blocking solution, and the IgG conjugation mixture containing biotin conjugated IgG, PNGase F, mTG, and excess biotin linker was added to the magnetic beads. The protein G magnetic beads incubated for 1 h in an automated vortex mixer at 1300 rpm to bind the IgG. After IgG capture, the magnetic beads were washed to remove all unbound proteins and reagents. The beads were washed three times with 500 μL PBS-T (0.05% Tween-20) for 5 min while mixing at 1300 rpm. To elute the IgGs, the magnetic beads were resuspended in 40 μL of elution buffer (10 mM glycine HCl pH 1.5) and allowed to incubate for 5 min in the vortex mixer at 1300 rpm. After elution, the tube was placed into the magnetic stand and the supernatant, containing isolated IgGs, was transferred to a new tube containing 10 μL neutralization buffer (2 M Tris buffer pH 7.5). Isolated IgGs were mixed gently into the neutralization buffer by pipet.

#### Random

Antibodies were randomly biotinylated with an activated NHS ester biotin linker. In a low binding tube containing 1 mg/mL antibody in PBS, NHS-PEG_4_-biotin solution was added to achieve a 40-fold excess of biotin to antibody. The reaction mixture was incubated at room temperature for 1 h. Immediately following incubation, excess biotin linker was removed from the solution by purification with the protein G magnetic beads following the same procedure as the site-specific biotinylated antibodies.

### Western Blot of Biotin-Antibody Conjugates

To visualize the biotinylated protein fragments, Western blots were performed on site-specific and random biotinylated antibodies. After electrophoresis, the SDS-PAGE gel soaked in freshly prepared transfer buffer (192 mM glycine, 24 mM Tris base, and 20% methanol), and the PVDF membrane soaked in methanol for 10 min on a rocker. The membrane sandwich was assembled as follows: sponge pad, filter paper, SDS-PAGE gel, PVDF membrane, second filter paper, second sponge pad. The membrane sandwich, an ice block, and a magnetic stir bar were placed in a tank of transfer buffer. Under constant stirring, protein transfer electrophoresed at 100 V for 1 h. After electrophoresis, empty space of the PVDF membrane was blocked with a 10% nonfat dry milk solution in tris-buffered saline (TBS) pH 7.4 (100 mM Tris base, 137 mM NaCl, and 2.7 mM KCl) for 1 h on a rocker. After blocking, the membrane was washed three times, each wash consisting of approximately 25 mL TBS with 0.1% Tween-20 (TBS-T) that incubated on the rocker for 5 min. Next, the membrane incubated with a streptavidin-horseradish peroxidase conjugate (SA-HRP) diluted 1:20,000 in TBS-T with 1% nonfat dry milk. The washed membrane was immersed in 20 mL of the SA-HRP solution for 1 h on the rocker. The membrane was then washed three times with 25 mL aliquots of TBS-T, three times with 25 mL aliquots of TBS, and once with 25 mL deionized water. Each wash step was incubated for 5 min on the rocker. Detection of bound SA-HRP was visualized by immersing the membrane in 20 mL of 1-Step Ultra TMB equilibrated to room temperature before use. Detection was closely monitored, and color development was stopped by two deionized water washes in quick succession.

### HABA-Avidin Quantitation of Biotin per Antibody

The number of biotin moieties conjugated to each IgG molecule was quantified with a HABA-avidin assay. A solution of the HABA-avidin complex consisted of 0.5 mg/mL avidin and 0.3 mM HABA in pH 7.2 phosphate buffer (100 mM). The number of biotin molecules per IgG was quantified from the change in absorbance at 500 nm of the HABA-avidin complex before and after the introduction of a biotinylated sample. An initial absorbance of 90 μL of the HABA-avidin solution was measured at 500 nm, then 10 μL of purified biotinylated IgG was added directly into the cuvette of HABA-avidin solution, mixed gently, and the absorbance at 500 nm was remeasured. The number of biotin molecules per IgG was calculated using the change in absorbance at 500 nm as well as the concentration of antibody.

### Antigen Capture Assay

Streptavidin-coated 96-well plates were used to assess the antigen capture ability of site-specifically and randomly biotinylated anti-HRP antibodies. Wells were prepared by triple-washing with 150 μL aliquots of PBS-T, then 100 μL of 200 nM biotinylated antibody was added to duplicate wells. The plate was covered with parafilm then incubated at 4 °C overnight. Excess and unbound antibody was then removed for subsequent analysis using a BCA protein assay to quantify excess antibody, and the plate was triple-washed with 150 μL aliquots of PBS-T. HRP antigen was diluted with PBS-T to concentrations of 0, 0.1, 1, 5, 10, 50, 100, and 200 nM, then 100 μL of each concentration was added to duplicate antibody functionalized wells. The plate was covered with parafilm then incubated at room temperature for 30 min. Excess and unbound HRP antigen was removed by triple-washing with 150 μL aliquots of PBS-T. Room temperature 1-Step ABTS (150 μL) was added to each well, and the rates of HRP-catalyzed ABTS oxidation were spectrophotometrically measured at 410 nm with a Thermo Varioskan LUX plate reader.

## Results and Discussion

A short synthetic peptide (e.g., KKCC or KKKCCC) was selected as the chemical modifier to systematically evaluate mTG for site-specific conjugation to IgGs from multiple host species. mTG can recognize the peptide N-terminus and a lysine side chain to conjugate the peptide onto an IgG. Moreover, it has been suggested that deglycosylation of IgG is not necessary when coupling a lysine modifier to an IgG via mTG.^54^ Tailored peptide sequences are readily accessible from commercial sources at a relatively low-cost and available with a fluorescent tag. Conjugation of a fluorescently labeled peptide serves the purpose of easily confirming site-specific conjugation, and although not the objective of this work, it could potentially function as a suitable fluorescently labeled antibody for use as a detection antibody in an immunoassay. Here, the objective is to directionally immobilize an antibody on a gold surface, and the peptide without the fluorescent dye is hypothesized to facilitate oriented immobilization on gold substrates (e.g., sensor surface and gold nanoparticles) that are integral to many established and novel biosensors. The peptide is designed to spatially localize multiple cysteine residues to provide protein attachment through multiple Au–S interactions^55–58^ and lysine residues to provide a high density of positive charge that will govern the direction at which the antibody approaches the gold surface to facilitate proper orientation.^59–64^ Thus, based on this rationale, we expected to demonstrate site-specific installation of dansyl-KKKCCC (e.g., fluorescent tag) on the IgG F_c_ region and the site-specific conjugation of KKKCCC (e.g., immobilization reagent) on the IgG F_c_ region for oriented immobilization as a model capture antibody.

### Site-Specific Conjugation of Fluorescently Labeled Peptide to IgGs

Human IgG2 was first evaluated for site-specific conjugation of the fluorescent peptide via mTG. mTG-mediated conjugation of drug molecules to human IgG2 has been extensively studied, and here it serves as a positive control benchmark to confirm dansyl-KKKCCC is a suitable substrate for mTG. An overview of the conjugation process is illustrated in Figure 1. Human IgG2 is first deglycosylated via PNGase F, followed by installation of the peptide with mTG. The conjugate is then fully reduced into light and heavy chains for electrophoretic separation, where only the IgG fragments conjugated to the peptide are observed in a fluorescent image. Figure 2 presents the SDS-PAGE results from the conjugation experiment. Figure 2A (lane 6) establishes that only the heavy chain that includes the privileged Q295 residue is visible in the fluorescent image. The corresponding Coomassie stained image in Figure 2B (lane 6) confirms the presence of both the heavy and light chains of the reduced IgG, as well as mTG (∼38 kDa) and a faint band for PNGase F (∼36 kDa). Site-specific conjugation was also observed for the IgG sample in which the deglycosylation step was omitted (e.g., no PNGase F), although the fluorescence intensity was reduced indicating that the conjugation was less efficient in the presence of the native glycan (Figure 2A, lane 4). While this supports the claim that deglycosylation is not necessary for lysine-containing substrates,^54^ data also reflect the clear benefit to this additional step.^48^ Additional controls validate the essential role of mTG in mediating the site-specific conjugation of the peptide to IgG (Figure 2A,2B, lanes 3 and 5).

**Figure 1:**
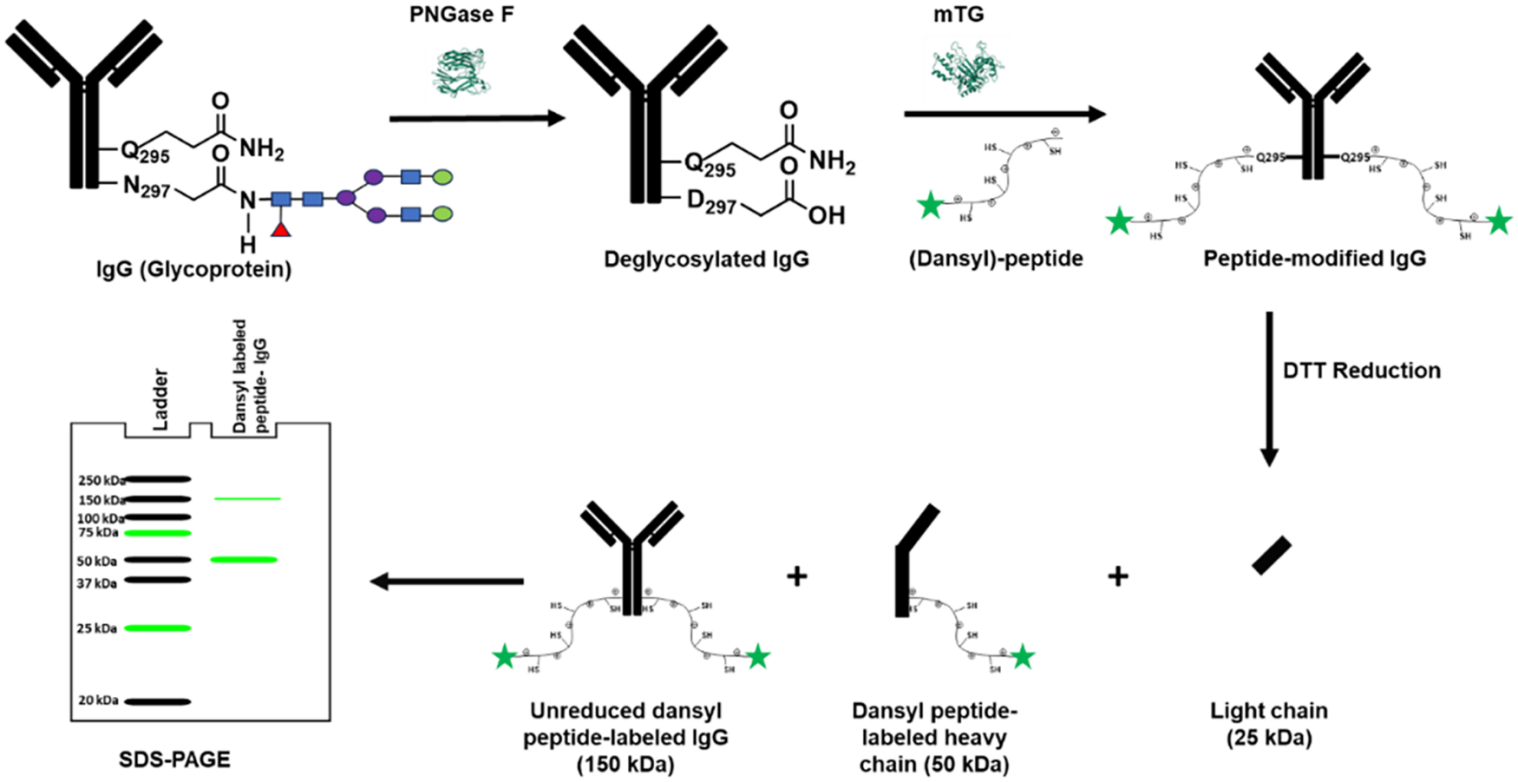
Workflow of peptide-IgG conjugation via mTG. Deglycosylation of IgG using PNGase F in the first step followed by mTG-mediated conjugation of dansyl-labeled peptide to the deglycosylated IgG, DTT reduction, and SDS-PAGE showing generated bands.

**Figure 2:**
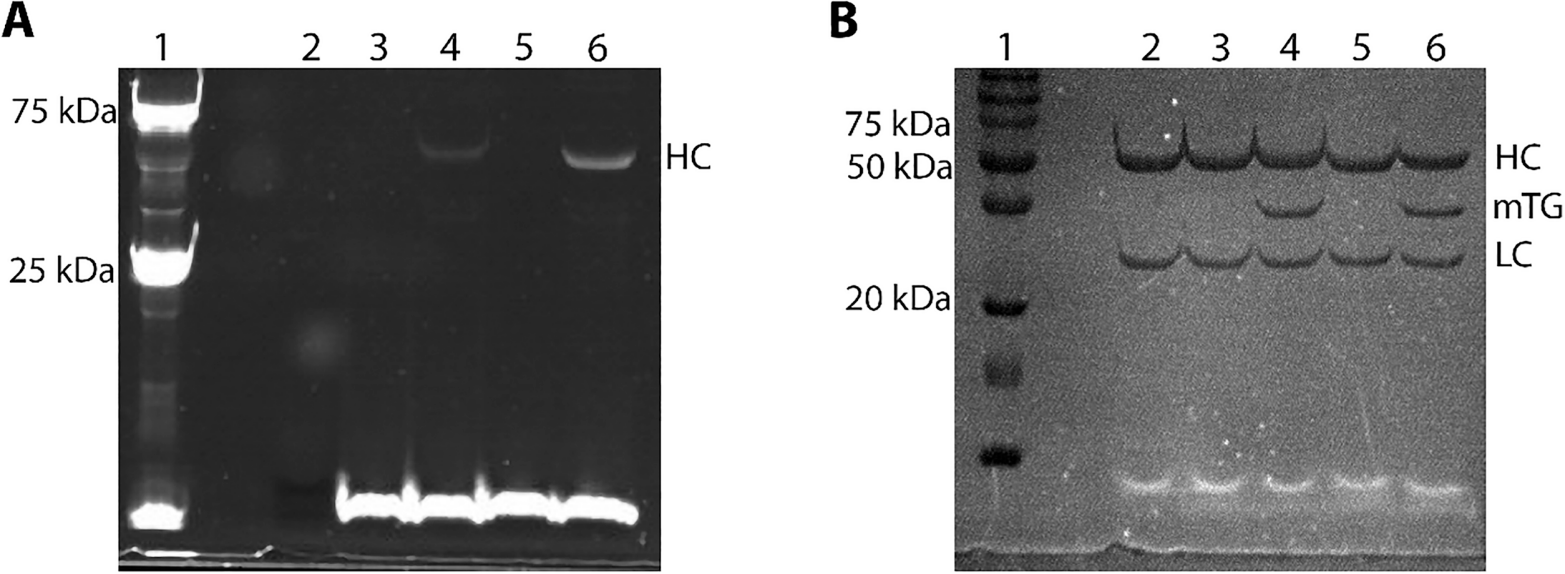
SDS-PAGE results for mTG mediated conjugation of peptide dansyl-KKKCCC to Human IgG2. (A) UV image generated at 365 nm from the gel imager. (B) Image generated from the gel imager after overnight Coomassie staining and lamination of the resultant gel. Lane 1: molecular weight standards ladder, lane 2: native human IgG2, lane 3: native human IgG2 + dansyl-KKKCCC, lane 4: native human IgG2 + dansyl-KKKCCC + mTG, lane 5: deglycosylated human IgG2 + dansyl-KKKCCC, lane 6: deglycosylated human IgG2 + dansyl-KKKCCC + mTG.

After confirming dansyl-KKKCCC is a suitable substrate for mTG and deglycosylation enhances conjugation efficiency, the range of IgGs that can be site-specifically modified was investigated. The Q295 is conserved on all human IgGs, rat IgG1, and mouse IgG1, based on amino acid sequence alignment (Figure S1), and it is anticipated that mTG operates to site-specifically modify all antibodies of these types. Figure 3 presents the SDS-PAGE results for mTG mediated conjugation of peptide dansyl-KKCC to human IgG1, human IgG2, rat IgG1, and mouse IgG1. The heavy chain of all tested IgGs were visible in the fluorescent image, while the light chain was not observed, providing evidence for site-specific conjugation of the deglycosylated native IgGs. It is noted that the peptide was shortened to highlight conjugation independence with respect to peptide length, and the site-specific modification results for human IgG2 are equivalent for dansyl-KKKCCC (Figure 2) and dansyl-KKCC (Figure 3). Like human IgG2, previous reports have established chemo-enzymatic conjugation of human IgG1 using mTG; however, there is limited evidence for mTG-mediated conjugation to rat IgG1 and mouse IgG1. This result is of particular significance because many biosensors and diagnostic assays rely on rat IgG1 and mouse IgG1 monoclonal antibodies for antigen capture and detection with high affinity and specificity. The versatility and scope of mTG-mediated coupling was further explored with the conjugation to polyclonal IgGs from rabbit and goat. These host species are frequent donors from which polyclonal antibodies are harvested and play a substantial role in bioassay applications. Amino acid sequence alignment (Figure S1) confirms Q295 is conserved in rabbit and goat IgGs, and Figure S2 reveals site-specific conjugation of the fluorescent peptide to these polyclonal IgG samples. Collectively, these data suggest a pathway to site-specifically couple reporter molecules responsible for signal transduction and cross-linking reagents responsible for immobilization that minimize interference with antigen binding for the prospective development of improved bioassays.

**Figure 3:**
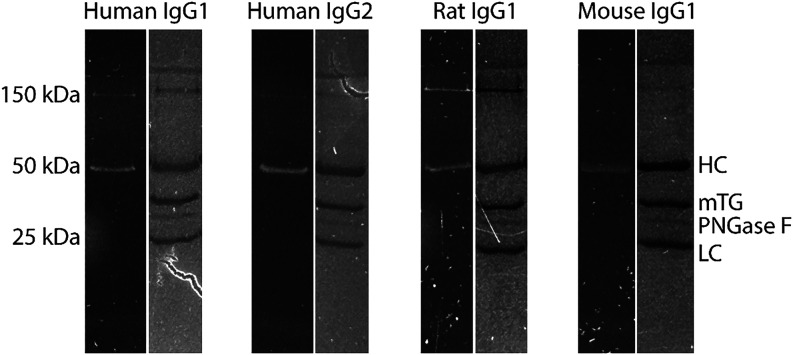
SDS-PAGE results for mTG mediated conjugation of peptide dansyl-KKCC to human IgG1, human IgG2, rat IgG1, and mouse IgG1. For each IgG sample, the left lane is the UV image to show successful mTG conjugation of the fluorescent peptide to the heavy chain, and the right lane is the Coomassie staining of the same gel to visualize all proteins and protein fragments present within the sample.

As discussed, the selected peptide is hypothesized to operate as a chemical linker to tether the F_c_ region of an IgG to a gold surface.^55–58^ Subsequent immobilization studies require a purification step to isolate the peptide-modified IgG from excess peptide, mTG, and PNGase F. To this end, a nonlabeled peptide (e.g., KKCC) was site-specifically installed on the human IgG1 and modified IgG was purified using protein G magnetic beads. The success of the magnetic bead purification step was monitored via SDS-PAGE, and the results are presented in Figure 4. SDS-PAGE clearly shows four bands in the conjugation mixture (Figure 4, lane 3) resulting from the IgG heavy chain, IgG light chain, mTG, and PNGase F. The purified IgG sample eluted from the protein G beads only generated two bands for the IgG heavy chain and IgG light chain (Figure 4, lane 4), confirming successful removal of mTG and PNGase F. Analysis of the supernatant from the protein G purification process shows two bands for the unbound mTG and PNGase F, and confirms sufficient capacity of the protein G beads to bind all IgG in the conjugation reaction mixture (Figure 4, lane 5). Recovery of the eluted IgG from the protein G beads yielded ∼40% of the initial IgG mass; therefore, BSA was added to the protein G magnetic beads in an effort to minimize any nonspecific binding of IgG to the beads and/or microcentrifuge tube and improve recovery. This gave a slight increase in IgG recovery (∼60–65%), with no observable BSA contamination in the purified peptide-IgG conjugate (Figure 4, lanes 6 and 7).

**Figure 4:**
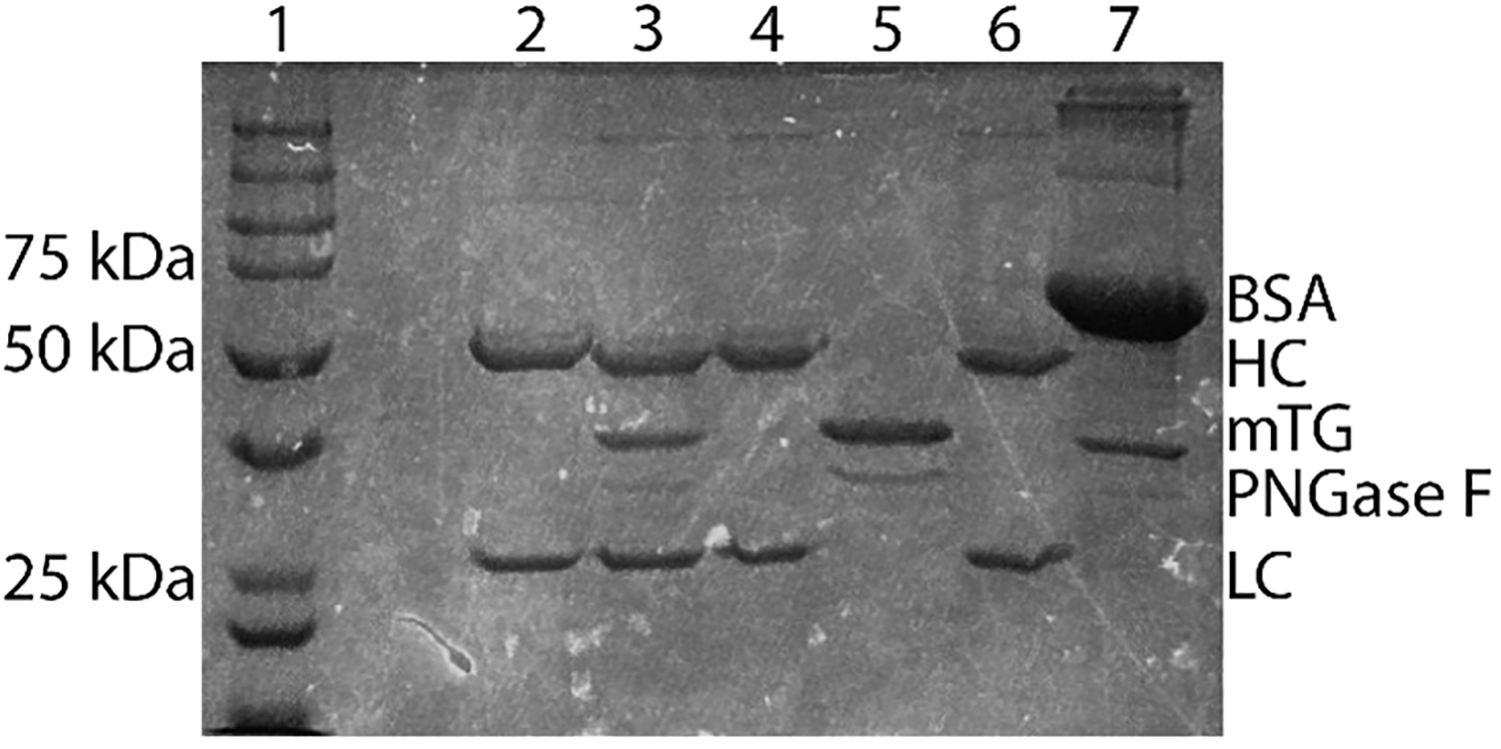
SDS-PAGE results for protein G magnetic beads for improved purification of peptide (KKCC)-modified human IgG1 via blocking agents (PBS vs BSA). Lane 1: molecular weight standards ladder, lane 2: native human IgG1, lane 3: unpurified human IgG1 + KKCC + PNGase F + mTG, lane 4: protein G purified human IgG1-(KKCC)_2_ with PBS (control), lane 5: unbound supernatant for human IgG1 purification with PBS (control), lane 6: protein G purified human IgG1-(KKCC)_2_ with BSA blocking, lane 7: unbound supernatant for human IgG1 purification with BSA blocking.

The purified peptide-IgG conjugate was then added to 60 nm citrate-capped gold nanoparticles (AuNPs) in a study designed to investigate the utility of the peptide modifier to facilitate robust and oriented chemisorption to the gold nanoparticle, in contrast to the random orientation observed for the adsorption of unmodified IgG onto gold nanoparticles.^65^ However, the AuNPs irreversibly aggregated immediately upon addition of the peptide-IgG conjugate at pH 7.4. This was attributed to electrostatic bridging, in which the additional positive charges installed on the IgG from protonated lysine residues in the peptide induces aggregation of negatively charged AuNPs.^59,66–68^ Attempts to reduce the positive charge through deprotonation at increased pHs, without denaturing the antibody, were not successful at preventing AuNP aggregation.^59,68^ Consequently, developing capture antibodies by exploiting the physicochemical properties of the KKCC peptide for chemisorption was not feasible, and an alternative immobilization chemistry was required to assess mTG-mediated site-specific conjugation for oriented antibody immobilization and enhanced antigen binding properties.

### Site-Specific Conjugation of Biotin to IgG

Biotin was explored as an alternative to the peptide as a strategy for oriented IgG immobilization facilitated by site-specific conjugation to IgG heavy chain. Capture antibodies are often biotinylated, allowing for immobilization on a streptavidin functionalized surface. The biotin–streptavidin interaction is highly specific and robust, with an affinity dissociation constant of ∼10^–15^ M.^69^ Conventionally, IgG antibodies are biotinylated using chemical coupling (e.g., NHS analog of biotin) to reactive amines provided by lysine residues. This chemical modification process installs biotin groups at random locations throughout the IgG molecule to result in random orientations upon immobilization on streptavidin supports. Site-specific coupling of biotin to native, nonhuman antibodies to control orientation is highly desirable to potentially advance biosensor performance.

An NH_2_–PEG_4_-biotin was conjugated to human IgG1, human IgG2, rat IgG1, and mouse IgG1 using mTG, following the procedure established for site-specific conjugation of the dansyl-peptide modifier. Ideally, mTG should catalyze the coupling of the terminal amine of the biotin analog to the Q295 residue. In parallel, each of the IgGs was reacted with NHS-PEG_4_-biotin to randomly conjugate biotin to the IgGs, using established chemical conjugation conditions.^70,71^ A Western blot was performed to characterize the biotinylated IgGs and to evaluate the site-specificity of the modifications. Each of the conjugated IgGs was fully reduced and separated by SDS-PAGE. The protein fragments were transferred to a PVDF membrane and treated with an HRP-streptavidin complex to bind biotinylated protein fragments. Following TMB development, the biotinylated fragments were easily visualized. Biotin was conjugated only to the heavy chain of each IgG for the mTG-mediated conjugation (Figure 5). Conversely, biotin was incorporated into both the heavy and light chains of each IgG type for the chemical (e.g., random) conjugation (Figure 5). Corresponding SDS-PAGE gels stained with Coomassie blue verified the presence of the light chain in the reduced site-specific IgG conjugate sample (Figure S3).

**Figure 5:**
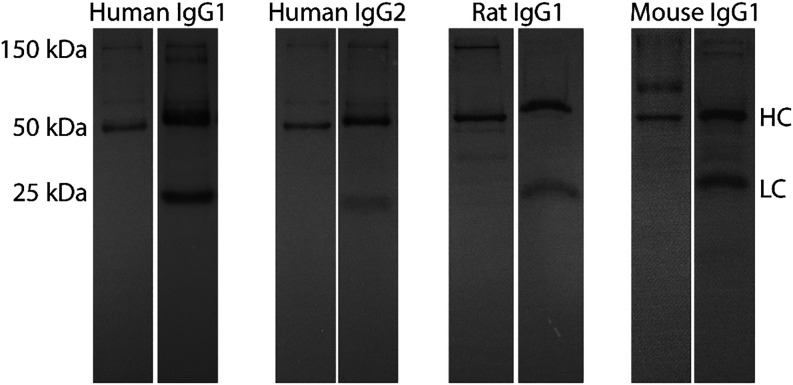
Western Blots for human IgG1, human IgG2, rat IgG1, and mouse IgG1. For each IgG represented, the left lane is site-specific biotinylated antibody (0.25 μg sample) and the right lane is random biotinylated antibody (0.25 μg sample).

The biotin-IgG conjugates were further characterized using a HABA-avidin assay to estimate the biotin-to-IgG ratio. If mTG is compatible with human IgG1, human IgG2, rat IgG1, and mouse IgG1, then enzyme-directed, site-specific biotinylation is expected to conjugate two biotin units per IgG molecule, one biotin to the Q295 on both heavy chains. In contrast, NHS-biotin reacts with the abundant surface accessible lysine residues and is anticipated to result in a biotin-to-IgG ratio greater than two, depending on the reaction time and pH. As anticipated, mTG-mediated conjugation of native, deglycosylated IgG resulted in ∼2 biotin units per IgG molecule (Table S1). In comparison, chemical biotinylation provided greater conjugation with most IgG types resulting in 6–8 biotins per IgG molecule (Table S1). These data further corroborate the versatility of mTG to site-specifically conjugate aminated reagents to IgG from a variety of host species. Moreover, these data confirm the successful synthesis of two biotinylated analogs of the same IgG, site-specific and random, to evaluate the feasibility of oriented immobilization for enhanced antigen binding capacity.

### Immobilization of Biotinylated Antibody and Antigen Capture

We selected anti-horseradish peroxidase antibodies (anti-HRP) for biotinylation and immobilization studies. Anti-HRP antibodies provide a convenient strategy to directly probe the orientation and antigen binding capacity upon immobilization on a solid substrate using a colorimetric assay.^65^ Specifically, immobilized antibody selectively binds HRP molecules from a sample solution; the captured HRP catalyzes the oxidation of a substrate (e.g., ABTS), and the reaction rate correlates with the number of captured HRP molecules. Additionally, monoclonal anti-HRP antibodies are commercially available as rat IgG1 and mouse IgG1, both isotypes and subclasses compatible with mTG-mediated conjugation as demonstrated above. A third anti-HRP antibody was also available and tested, belonging to the mouse IgG2b subclass; however, this was not anticipated to generate site-specific conjugates, because mouse IgG2b heavy chain does not have the required Q295 recognized by mTG (Figure S1).

Three strategies were implemented to immobilize antibody and perform comparative assays for antigen detection (Figure 6A). All assays were performed in a 96-well plate that was prefunctionalized with streptavidin to leverage the strong and specific interaction between biotin and streptavidin. Site-specific and random biotinylated anti-HRP antibodies were directly immobilized to the streptavidin-coated wells to form the capture antibody layer in two of the assay formats. This design allows us to elucidate oriented immobilization potentially achieved with site-specific biotinylation of the antibody. In a third assay design, biotinylated protein G was first immobilized onto the streptavidin-coated wells, followed by adsorption of anti-HRP antibodies. Protein G binds the F_c_ region of IgG and is routinely used to immobilize antibody in a preferred orientation.^16,17^ Thus, protein G-mediated immobilization of anti-HRP antibody serves as a benchmark to compare immobilization of mTG-mediated site-specific biotinylated anti-HRP capture antibody.

**Figure 6:**
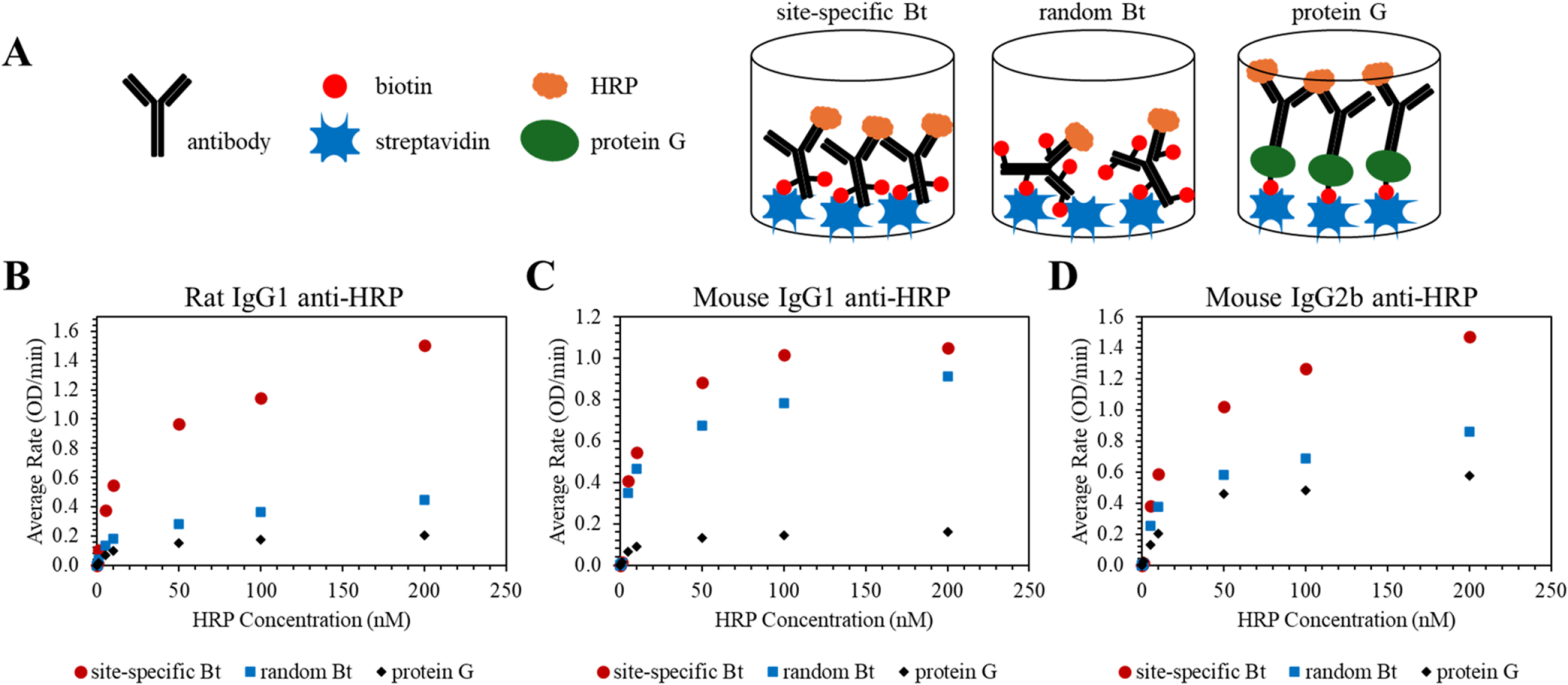
HRP capture assays comparing site-specific biotinylation, random biotinylation, and protein G immobilization. Immobilization scheme of the site-specifically biotinylated antibodies, randomly biotinylated antibodies, and biotinylated protein G oriented native antibodies in streptavidin-coated wells (A). Dose-dependent HRP binding curves for rat IgG1 anti-HRP antibodies (B), mouse IgG1 anti-HRP antibodies (C), and mouse IgG2b anti-HRP antibodies (D) represented as the rate of HRP-catalyzed ABTS oxidation.

The immunoassays for the detection of HRP using immobilized rat IgG1 anti-HRP antibody are presented in Figure 6B. The capture antibody layer was formed from a 200 nM solution to ensure consistent and saturated coverage of antibody in the streptavidin functionalized well and maximize the number of antigen-binding sites (Figure S4). Varying concentrations of HRP were added to separate wells and incubated for 30 min to allow for binding. After thorough rinsing to remove excess unbound HRP, ABTS was added, and the rate of HRP-catalyzed oxidation of ABTS was measured spectrophotometrically to quantify bound HRP. All three immunoassay formats with the rat IgG1 anti-HRP antibody resulted in a dose–response curve, where the reaction rate (e.g., captured HRP) increased with increasing HRP concentration at low concentrations, and the signal plateaued at high concentrations where the antigen binding sites presented by the immobilized antibody saturated. Analysis to best fit the data in Figure 6 to a ligand binding curve revealed maximum rates of 1.50 OD/min and 0.44 OD/min for site-specific and random biotinylated antibody, respectively. At each antigen concentration the signal obtained for the site-specific biotinylated antibody exceeds that of the random biotinylated antibody by ∼3-fold (Table S2). Interestingly, oriented immobilization of rat IgG1 antibody through protein G provided less signal (e.g., less HRP capture) than random biotinylation, with a maximum rate at saturating antigen concentrations of 0.20 OD/min from a best fit analysis.

The surface concentration of immobilized antibodies was quantified to further understand the differences in antigen binding observed in Figure 6. A BCA total protein assay was used to quantify the excess antibody remaining in solution after allowing for antibody binding in the streptavidin-coated wells, and the immobilized surface concentration was calculated as the difference between the amount of antibody added to the well and the excess unbound antibody (Figure S5). Notably, equivalent surface loading was achieved for site-specific (1.8 ± 1.0 pmol/well) and random (2.3 ± 0.2 pmol/well) biotinylated antibody, suggesting differences in antigen binding is due to orientation. This is consistent with previous reports concluding that ∼25% of antigen binding sites are accessible when immobilization results in random orientation of antibody.^65,72^ Moreover, the antibody surface loading using a protein G surface (0.22 pmol/well) was approximately 8× lower than that of the site-specific biotinylated antibody; thus, the antigen:antibody binding ratio is equivalent for the two systems when normalized to surface density of antibody. This further supports the conclusion that mTG-mediated modification of antibody leads to a proper orientation of the antibody (i.e., equivalent to the established protein G benchmark). Together, the combined contributions of antibody orientation and surface concentration are responsible for the improved functional assay achieved by immobilization of the site-specific biotinylated antibody.

The same three immobilization strategies were used to evaluate immunoassays for HRP detection using the mouse IgG1 anti-HRP monoclonal antibody (Figure 6C). The trend in assay performance with respect to immobilization method was similar to that of rat IgG1. Site-specific immobilization gave the most sensitive binding curve and lowest limit of detection, although the improvement relative to the randomly biotinylated antibody was less pronounced with only ∼1.25-fold signal enhancement (Table S3). Quite surprisingly, mouse IgG2b anti-HRP conjugates gave the same trend in immunoassay performance for the three assay configurations (Figure 6D and Table S4). An antigen binding curve was expected for immobilization of this randomly biotinylated antibody, because surface accessible lysine residues are available on mouse IgG2b for conjugation of biotin via chemical modification to install biotin moieties. Moreover, protein G mediated immobilization of this antibody was expected due to documented affinity of protein G to mouse IgG2b. However, the Q295 residue recognized by mTG is not conserved in mouse IgG2b; therefore, biotin was not expected to be installed on the antibody labeled as “site-specific”. Nevertheless, mTG facilitated the conjugation of NH_2_–PEG_4_-biotin to this mouse IgG2b anti-HRP antibody, and immobilization of this antibody conjugate to the streptavidin functionalized surface provided the greatest antigen binding performance.

To better understand this result, we first confirmed that the mouse IgG2b anti-HRP antibody was correctly classified using a mouse IgG isotyping kit (Figure S6). Next, we used mTG to conjugate the fluorescent peptide to a mouse IgG2a isotype control, mouse IgG2b isotype control, and the mouse IgG2b anti-HRP antibody used in the antigen binding assay. The IgGs were electrophoresed under reducing conditions and imaged. The fluorescent images confirm that mTG did not conjugate the peptide to mouse IgG2a while mTG conjugated the peptide to the light chain of the mouse IgG2b isotype control and the heavy chain of the mouse IgG2b anti-HRP antibody (Figure S7). The mouse IgG2b isotype control and mouse IgG2b anti-HRP antibody were further investigated by mTG conjugation of biotin and characterized by a Western blot (Figure S8). The Western blot confirms biotin was chemically conjugated to the light chain of the mouse IgG2b isotype control and the heavy chain of the mouse IgG2b anti-HRP antibody, corroborating results for mTG-mediated conjugation of the fluorescent peptide substrate. Recently, Schibli and Spycher reported that mTG conjugated an amine substrate to a small fraction (∼7%) of mouse IgG2b heavy chains and ∼96% of mouse IgG2b light chains, while no conjugation was detected to the light nor heavy chains of mouse IgG2a.^73^ These results for the mouse IgG2a and IgG2b isotype controls are highly consistent with those recently reported, and indicate that the mouse IgG2b anti-HRP antibody falls within the small fraction (7%) of mouse IgG2b that are modified on the heavy chain by mTG. Importantly, the mTG-direct modification sites on mouse IgG2b are not currently known. Regardless of the specific modification site, the modification is heavy chain specific for mouse IgG2b anti-HRP antibody and resulted in greater antigen binding in a functional assay.

Collectively, these data establish mTG efficiently and effectively conjugate an amino-biotin reagent to several IgG subclasses common to commercial antibody production. Moreover, site-specific installation of an immobilization agent resulted in increased antigen binding in a functional assay compared to more conventional immobilization techniques. The advantage of oriented antibody immobilization has been extensively documented,^14–18^ which has driven ongoing efforts to realize this controlled surface architecture. However, few reliable and robust methods have been developed for native antibodies, and those that have been explored exhibit varying degrees of success based on antibody subclass.^17,33,74,75^ mTG-directed conjugation offers the distinct advantage of versatility to site-specifically modify off-the-shelf, native antibodies isolated from several host species and a variety of IgG subclasses.

## Conclusions

We have described the scope of mTG as a chemo-enzymatic approach for the site-specific modification of IgGs from several host species and subclasses that are critical to the development of immunoassays and biosensors. mTG catalyzed the covalent coupling of amine reagents to the Q295 conserved in the heavy chain of many IgGs with stoichiometric specificity and high efficiency. We successfully installed a fluorescently labeled peptide, dansyl-KKCC, and an immobilization reagent, NH_2_–PEG_4_-biotin, as examples of chemical modifiers for use as detection antibodies and capture antibodies. Site-specific installation of the fluorescent peptide was confirmed by SDS-PAGE and biotinylation was verified by Western blots. Most significantly, we demonstrated the site-specific installation of these modifiers on several types of IgGs, including human IgG1, human IgG2, rat IgG1, mouse IgG1, and polyclonal IgGs from rabbits and goats. Our results established that the mTG-mediated biotinylation approach to antibody immobilization was superior to chemical (e.g., random) biotinylation and protein G mediated immobilization in a functional assay for antigen binding. The versatility of this conjugation strategy is a powerful approach for the production of highly functional labeled detection antibodies and immobilized capture antibodies for use in immunosensors.

## Supplementary Material



## Notes

Experimental details for HABA-Avidin assay and optimization of antibody immobilization; amino acid sequence alignment (Figure S1); SDS-PAGE for polyclonal IgGs (Figure S2); SDS-PAGE for biotinylated IgGs (Figure S3); saturation curve for antibody immobilization on streptavidin plates (Figure S4); BCA calibration curve (Figure S5); LFA for IgG isotyping (Figure S6); SDS-PAGE for mouse IgGs (Figure S7); Western blot for biotinylation of mouse IgG2b samples (Figure S8); biotin-IgG ratio for conjugates (Table S1); raw data for HRP capture assays (Tables S2–S4) (PDF)
