# Supplementary material for: Scope of Microbial Transglutaminase for Site-Specific and Oriented Immobilization of Native Antibodies from Various Host Species
Source: Langmuir. 2026 Feb 27;42(9):6903–13. doi: 10.1021/acs.langmuir.5c06485 (PMC12980840; doi:10.1021/acs.langmuir.5c06485)
Supplement: Supplementary file 1 [file la5c06485_si_001.pdf]

Supporting Information for:

Scope of microbial transglutaminase for site-specific and oriented immobilization of native antibodies from various host species

*Emily Beitello,[†] Kwame Osei,[†] Faith E. Breausche, Jon A. Friesen, and Jeremy D. Driskell**

Department of Chemistry, Illinois State University, Normal, IL USA 61790

[†]*Authors contributed equally*

HABA-Avidin Quantitation of Biotin per Antibody. The number of biotin moieties conjugated to each IgG molecule was quantified with a HABA-avidin assay. A solution of the HABA-avidin complex consisted of 0.5 mg/mL avidin and 0.3 mM HABA in pH 7.2 phosphate buffer. The number of biotin molecules per IgG was quantified from the change in absorbance at 500 nm of the HABA-avidin complex before and after the introduction of a biotinylated sample. An initial absorbance of 90 µL of the HABA-avidin solution was measured at 500 nm, then 10 µL of purified biotinylated IgG was added directly into the cuvette of HABA-avidin solution, mixed gently, and the absorbance at 500 nm was remeasured. The number of biotin molecules per IgG was calculated using the change in absorbance at 500 nm as well as the concentration of antibody.

Antibody Immobilization in 96-well Plate. Streptavidin-coated 96-well plates were used to assess the antigen capture ability of site-specifically and randomly biotinylated anti-HRP antibodies. The concentration of antibody needed to fully saturate a streptavidin coated well was determined by testing a range of antibody concentrations exposed to an excess of HRP antigen. Wells were prepared by triple-washing with 150 µL aliquots of PBS-T. Antibody samples were diluted with PBS-T to concentrations of 0, 1, 5, 10, 50, 100, and 200 nM then 100 µL of each concentration was added to duplicate wells. The plate was covered with parafilm then incubated at 4 °C overnight. Excess and unbound antibody was then removed by triple-washing with 150 µL aliquots of PBS-T. Antigen capture was then performed by adding 100 µL of 1000 nM HRP to all wells. The plate was covered with parafilm then incubated at room temperature for 30 min. Excess and unbound HRP antigen was removed by triple-washing with 150 µL aliquots of PBS-T. Finally, 150 µL of room temperature 1-Step ABTS was added to each well and the rate of HRP-catalyzed ABTS oxidation was spectrophotometrically measured at 410 nm with a Thermo Varioskan LUX plate reader.

	295 ↓
Human IgG1	PREE**Q**YNSTY
Human IgG2	PREE**Q**FNSTF
Rat IgG1	PREQ**Q**YNSTY
Mouse IgG1	PREE**Q**FNSTF
Mouse IgG2a	THRE**D**YNSTL
Mouse IgG2b	THRE**D**YNSTI
Rabbit IgG	LREQ**Q**FNSTI
Goat IgG	PREE**Q**FNSTF

Figure S1. BLAST multiple sequence alignment for IgG heavy chain representative of several host species and subtype, highlighting the amino acid at position 295.

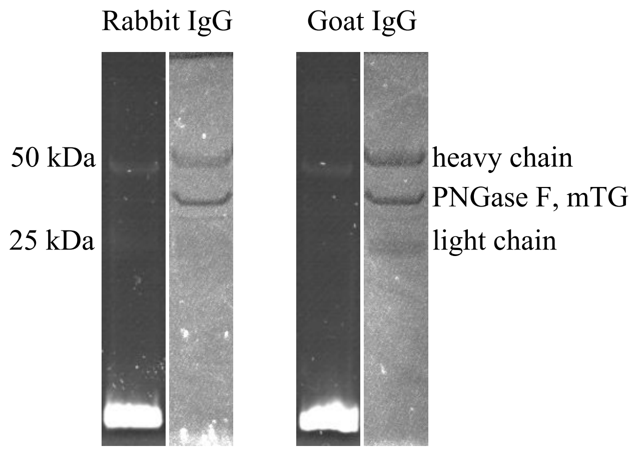

Figure S2. SDS-PAGE results for mTG mediated conjugation of peptide dansyl-KKCC to rabbit IgG and goat IgG. For each IgG sample the left lane is the UV image to show successful mTG conjugation of the fluorescent peptide to the heavy chain, and the right lane is the Coomassie staining of the same gel to visualize all proteins and protein fragments present within the sample.

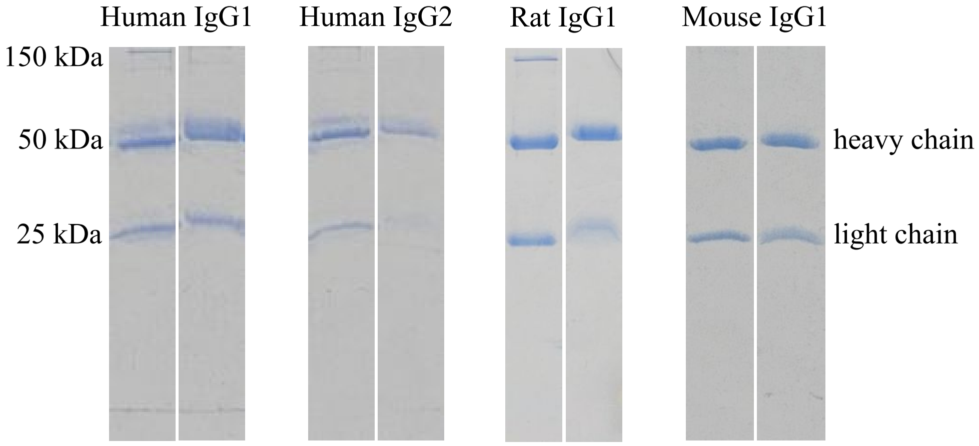

Figure S3. SDS-PAGE results for mTG mediated site-specific mTG conjugation of NH_2-PEG_4-biotin (left) and random chemical conjugation of NHS-PEG_4-biotin to human IgG1, human IgG2, rat IgG1, and mouse IgG1.

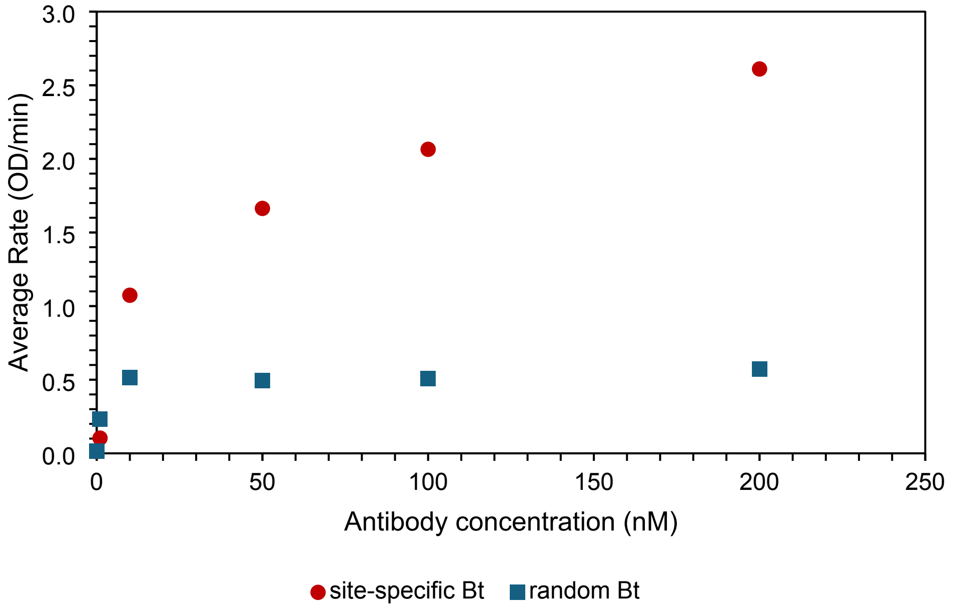

Figure S4. Antigen binding as a function of antibody concentration. The site-specific and random biotinylated anti-HRP antibodies were immobilized on a streptavidin functionalized 96-well plate at varying concentrations. HRP (1000 nM) was added to the wells to saturate available sites presented by the antibody. The bound HRP was quantified by the rate at which ABTS was oxidized.

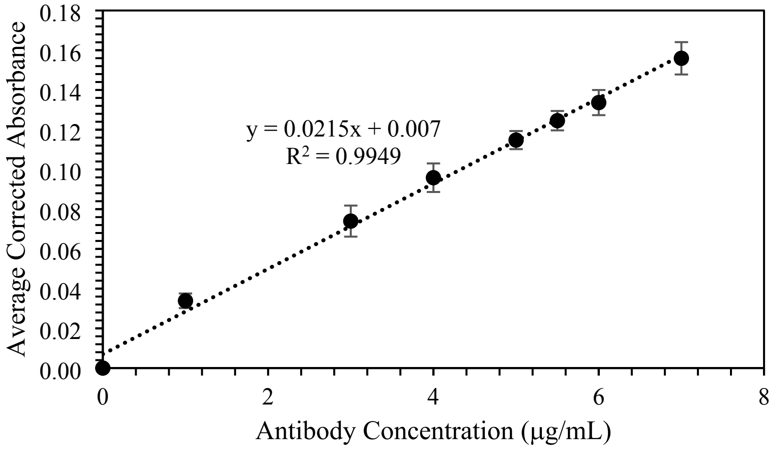

Figure S5. Average calibration curve for BCA total protein assay to quantify protein loading in 96-well streptavidin coated plate. A total of 7 independent calibration standards were prepared and analyzed to generate the data. Error bars represent the standard deviations.

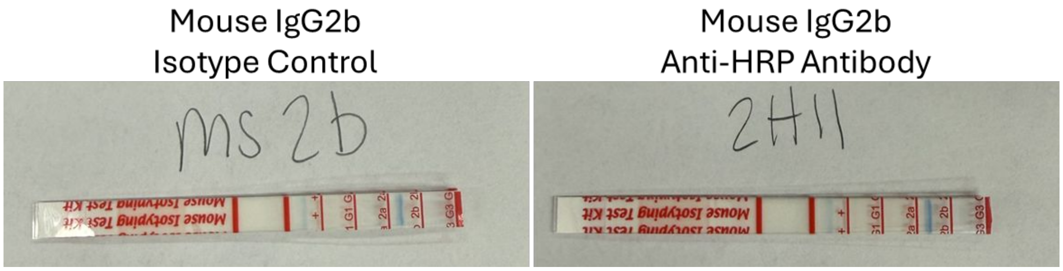

Figure S6. Photograph of lateral flow assay to test the isotype of a mouse IgG2b isotype control sample and mouse IgG2b anti-HRP antibody.

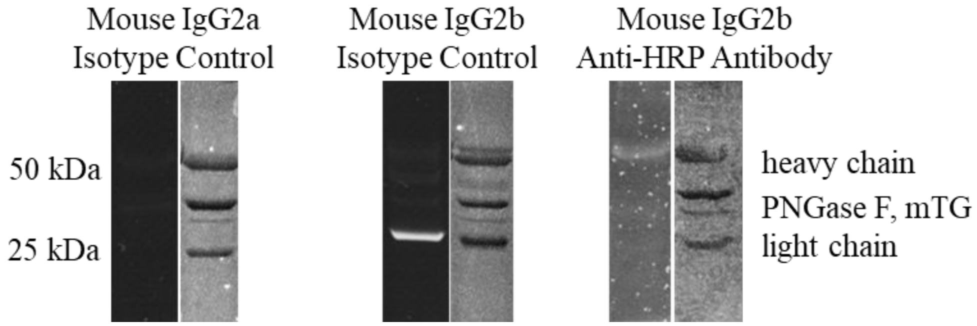

Figure S7. SDS-PAGE results for mTG mediated conjugation of peptide dansyl-KKCC to mouse IgG2a isotype control, mouse IgG2b isotype control, and mouse IgG2b anti-HRP antibody. For each IgG sample, the left lane is the UV image to visualize mTG conjugation of the fluorescent peptide to the heavy or light chains, and the right lane is the Coomassie staining of the same gel to visualize all proteins and protein fragments present within the sample.

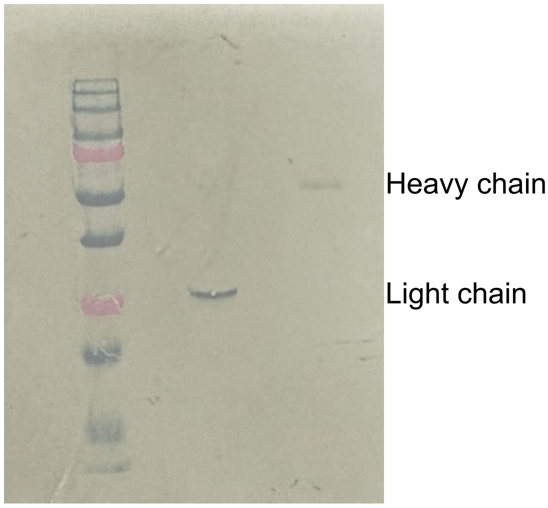

Figure S8. Western blot to stain biotinylated protein fragments. NH_2-PEG_4-biotin was conjugated to deglycosylated IgG samples using mTG. Lane 1: prestained molecular weight standards ladder, Lane 2: mouse IgG2b isotype control (2.5 μg sample), Lane 3: mouse IgG2b anti-HRP antibody (2.5 μg sample).

Table S1. The number of biotin units conjugated to each IgG molecule for site-specific (mTG) and random (chemical) conjugation measured using a HABA-avidin assay.

Sample	Site-specific Bt:Ab	Random Bt:Ab
Human IgG1	2.3	8.1
Human IgG2	1.7	7.7
Mouse IgG1	1.4	6.7
Mouse IgG1 anti-HRP (clone HP-03)	2.4	3.7
Rat IgG1 anti-HRP (clone HRPN)	2.3	6.1

Table S2. The rate of HRP-catalyzed ABTS oxidation measured for HRP binding assays conducted by immobilization of site-specific and random biotinylated rat IgG1 anti-HRP antibodies.

HRP Concentration (nM)	Site-specific Bt-Ab Rate (OD/min)	Random Bt-Ab Rate (OD/min)	Ratio (Site-specific:Random)
200	1.508	0.450	3.35
100	1.144	0.365	3.13
50	0.969	0.279	3.47
10	0.548	0.179	3.06
5	0.378	0.133	2.85
1	0.106	0.040	2.64
0.1	0.013	0.005	2.54
0	0.000	0.000	---

Table S3. The rate of HRP-catalyzed ABTS oxidation measured for HRP binding assays conducted by immobilization of site-specific and random biotinylated mouse IgG1 anti-HRP antibodies.

HRP Concentration (nM)	Site-specific Bt-Ab Rate (OD/min)	Random Bt-Ab Rate (OD/min)	Ratio (Site-specific:Random)
200	1.050	0.913	1.15
100	1.017	0.783	1.30
50	0.882	0.673	1.31
10	0.545	0.467	1.17
5	0.407	0.347	1.17
1	0.013	0.012	1.12
0.1	0.002	0.002	1.32
0	0.000	0.000	---

Table S4. The rate of HRP-catalyzed ABTS oxidation measured for HRP binding assays conducted by immobilization of site-specific and random biotinylated mouse IgG2b anti-HRP antibodies.

HRP Concentration (nM)	Site-specific Bt-Ab Rate (OD/min)	Random Bt-Ab Rate (OD/min)	Ratio (Site-specific:Random)
200	1.472	0.861	1.71
100	1.265	0.686	1.84
50	1.021	0.580	1.76
10	0.587	0.374	1.57
5	0.382	0.252	1.52
1	0.013	0.009	1.49
0.1	0.002	0.001	1.50
0	0.000	0.000	---
